# Angiographic Subtypes of Neovascular Age-related Macular Degeneration in Korean: A New Diagnostic Challenge

**DOI:** 10.1038/s41598-019-46235-3

**Published:** 2019-07-04

**Authors:** Kunho Bae, Sung Rae Noh, Se Woong Kang, Eung Suk Kim, Seung-Young Yu

**Affiliations:** 1 Department of Ophthalmology, Dongguk University, Ilsan Hospital, Goyang, South Korea; 2 Department of Ophthalmology, Kyung Hee University Hospital, Kyung Hee University, Seoul, South Korea; 3 Department of Ophthalmology, Samsung Medical Center, Sungkyunkwan University School of Medicine, Seoul, South Korea

**Keywords:** Retinal diseases, Diagnosis

## Abstract

Neovascular age-related macular degeneration (AMD) is the leading cause of irreversible blindness in elderly population. Several classifications schemes have been developed to provide subtypes of neovascular AMD, which are known to be associated with visual prognosis. However, there is still a large proportion of patient with ambiguous findings according to current classification criteria. In this study, we classified treatment-naïve neovascular AMD patients using novel angiographic classification system and investigated the incidence and clinical characteristics of AMD subtypes. Among 339 eyes, five AMD subtypes were identified: 41 (12.1%) with classic choroidal neovascularization (CNV), 30 (8.8%) with occult CNV, 91 (26.8%) with microaneurysmal choroidal vasculopathy (MCV), 123 (36.3%) with polypoidal choroidal vasculopathy (PCV), and 54 (15.9%) with retinal angiomatous proliferation (RAP). MCV was younger than RAP (*P* < 0.001). Classic CNV presented with worse visual acuity compared with MCV at baseline (*P* < 0.001). Central macular subfield thickness was highest in RAP, and lowest in MCV (*P* = 0.036). Subfoveal choroidal thickness was highest in MCV, and lowest in RAP (*P* < 0.001). There was a significant difference in visual acuity at 12 months among five subtypes (*P* = 0.046). Our results highlight the importance of angiography for identifying AMD subtypes, particularly the novel MCV group being distinct from other subtypes.

## Introduction

Age-related macular degeneration (AMD) is a progressive chronic disease of the central retina and a leading cause of vision loss worldwide^1^. There have been considerable advances in the diagnosis of AMD since Novotony and Alvis first introduced the use of fluorescein angiography (FA) in the human fundus^2^. Since then, several useful diagnostic systems have been introduced, including indocyanine green angiography (ICGA)^3^, optical coherence tomography (OCT)^4^, and, most recently, OCT angiography^5^. The advent of ICGA facilitated the detection and demarcation of occult or poorly defined choroidal neovascularization (CNV). ICGA allows retinal specialists to move beyond the present FA-based categorization of “occult/poorly defined” and “classic/well-defined” lesions to develop a more refined classification of CNV^6^. These new classifications include polypoidal choroidal vasculopathy (PCV)^7^ and retinal angiomatous proliferation (RAP)^8^.

The advent of intravitreal therapy using anti-vascular endothelial growth factor (VEGF)^9^ has introduced a new standard in the treatment of neovascular AMD patients, however, the major challenge associated with anti-VEGF treatment lies in the heterogeneity of individual patient profiles. Different types of CNV are different in terms of the demographic risk profile, clinical manifestations, visual prognosis and response to treatment^10,11^. In this regard, there is a need for more consensus regarding the classification system using angiography. More specifically, there is a portion of patients that fall on the boundaries of occult CNV and PCV^12^. This observation may explain why the prevalence of PCV is reported differently in studies conducted on patients of the same race (22.3–61.6%)^13^. Therefore, it is important to redefine the classification criteria using angiography, ultimately in order to refine present and future treatment strategies to improve visual outcomes.

Therefore, the aim of the present study was to determine the incidence and clinical characteristics of angiographic subtypes of neovascular AMD including microaneurysmal choroidal vasculopathy – a novel subtype of AMD that we hypothesized as a distinctive entity from PCV and occult CNV - and compare functional outcomes with other subtypes.

## Results

We identified 353 eyes from 304 patients with neovascular AMD based on their electronic records. Fourteen eyes were excluded because they had vitreous hemorrhages (n = 6), or because of an absence of ICGA photographs at baseline (n = 8). Ultimately, 339 eyes from 290 patients with neovascular AMD were included in this study. The mean age of the patients was 72.4 ± 8.0 years (range, 50~92), and 59.0% were male (Table 1). The most common types of neovascular AMD were PCV (36.3%) and MCV (26.8%). Classic CNV, occult CNV, and RAP were identified in 12.1%, 8.8%, and 15.9% of eyes, respectively. There was excellent interobserver reproducibility of the angiography-based classification (ICC = 0.804, 95% confidence interval 0.719–0.883). The patients in the MCV and PCV groups were younger than were those in the classic CNV or RAP groups. The proportion of men was significantly higher in the MCV and PCV groups than it was in the other groups (73.6% and 67.5%, respectively). At baseline, the BCVA in classic CNV, occult CNV, MCV, PCV, and RAP were 20/400, 20/100, 20/80, 20/100, and 20/142, respectively (P < 0.001). There were no significant differences across the five groups in the prevalence of subretinal hemorrhage at baseline.

**Table 1:** Baseline patient characteristics according to fluorescein angiography (FA) and indocyanine green angiography (ICGA)-based classification in neovascular age-related macular degeneration.

Characteristics n = 339	Classic CNV n = 41	Occult CNV n = 30	MCV n = 91	PCV n = 123	RAP n = 54	*P*- value
Age, yrs	76.8 ± 5.6 (62~87)	72.9 ± 6.9 (59~87)	69.7 ± 8.0 (50~84)	70.4 ± 7.8 (50~90)	77.9 ± 5.9 (65~92)	<0.001^a^
Sex (M/F)	22/19	14/16	67/24	83/40	14/40	<0.001^b^
Bilaterality	22 (55.0%)	15 (50.0%)	21 (23.1%)	30 (24.4%)	26 (48.1%)	<0.001^b^
BCVA, Snellen (logMAR)	20/400 (1.34)	20/100 (0.69)	20/80 (0.60)	20/100 (0.70)	20/142 (0.84)	<0.001^a^
CST, μm	287.8 ± 123.2 (110~547)	328.9 ± 108.0 (140~580)	298.4 ± 87.7 (108~557)	309.5 ± 87.5 (144~645)	348.9 ± 133.8 (190~884)	0.036^a^
Subretinal hemorrhage	5 (13.2%)	2 (6.7%)	12 (13.2%)	28 (22.8%)	9 (17.3%)	0.161^b^
**Type of FA**						<0.001^c^
Classic CNV	41 (100%)	0	15 (16.5%)	42 (34.1%)	7 (13.0%)	
Occult CNV	0	30 (100%)	76 (83.5%)	81 (65.9%)	30 (55.6%)	
RAP	0	0	0	0	17 (31.5%)	
**ICGA findings**						
Hot spot	2 (4.9%)	1 (3.3%)	69 (75.8%)	117 (95.1%)	41 (75.9%)	<0.001^b^
Branching vascular network	6 (14.6%)	2 (6.7%)	91 (100.0%)	104 (84.6%)	3 (5.6%)	<0.001^b^
Plaque	3 (7.3%)	12 (40.0%)	5 (5.5%)	4 (3.3%)	11 (20.4%)	<0.001^b^

CNV, choroidal neovascularization; MCV, microaneurysmal choroidal vasculopathy; PCV, polypoidal choroidal vasculopathy; RAP, retinal angiomatous proliferation; BCVA, best-corrected visual acuity; CST, central subfield thickness; FA, fluorescein angiography.

Continuous variables are reported as mean ± standard deviation (range) values. All other data are n (%).

^a^Calculated by one-way ANOVA.

^b^Calculated by chi-square test.

^c^Calculated by Kruskal-Wallis H test.

### Fluorescein angiography findings

Based on the FA findings, 105 eyes (31.0%) were suspected of having classic CNV and 234 eyes (69.0%) occult CNV. Those classified with occult CNV includes patients with occult with minimally classic CNV. Of the eyes with “classic CNV on FA,” 42 eyes (40.0%) were diagnosed with PCV, 41 (39.0%) with classic CNV, 15 (14.3%) with MCV, and 7 (6.7%) with RAP on ICGA. Of the eyes with “occult CNV on FA,” 81 eyes (34.6%) were diagnosed with PCV, 76 (32.5%) with MCV, 30 (12.8%) with occult CNV, and 47 (20.1%) with RAP on ICGA.

### Indocyanine green angiography findings

Eyes in the MCV, PCV, and RAP groups had a higher proportion of hot spots than did those in the other groups (75.8%, 95.9%, 75.9%, respectively, P < 0.001). Polyps were identified after there was pneumatic displacement of submacular hemorrhages in 5 eyes in the PCV group. In these cases, the polyps were not visible at baseline. BVN were detected in 14.6% of classic CNV, 6.7% of occult CNV, 100% of MCV, 84.6% of PCV, and 5.6% of RAP patients (P < 0.001). Plaques were detected in the late phase of ICGA in 40.0% of occult CNV and 20.4% of RAP cases, which differed significantly from those in the other groups (P < 0.001).

### Optical coherence tomography findings

At baseline, the CST in classic CNV, occult CNV, MCV, PCV, and RAP were 287.8 ± 123.2 *μm*, 328.9 ± 108.0 *μm*, 298.4 ± 87.7 *μm*, 309.5 ± 87.5 *μm*, and 348.9 ± 133.8 *μm*, respectively (P = 0.036). The subfoveal choroidal thickness in classic CNV, occult CNV, MCV, PCV, and RAP were 145.5 ± 85.1 *μm*, 183.5 ± 61.9 *μm*, 273.8 ± 105.3 *μm*, 232.8 ± 88.8 *μm*, and 125.4 ± 51.4 *μm*, respectively (P < 0.001, Table 2). Subretinal fluid was noted in 31.7% of classic CNV, 73.7% of occult CNV, 86.8% of MCV, 93.5% of PCV, and 42.6% of RAP eyes (P < 0.001). The proportion of subretinal fibrosis was prominent in the classic CNV group, which was also significantly different from that in the other groups (92.7%, P < 0.001).

**Table 2:** Spectral-domain optical coherence tomographic (OCT) characteristics of patients according to the fluorescein angiography and indocyanine green angiography-based classification in neovascular age-related macular degeneration.

Characteristics	Classic CNV n = 41	Occult CNV n = 30	MCV n = 91	PCV n = 123	RAP n = 54	*P*-value
Choroidal thickness, μm	145.5 ± 81.5	183.5 ± 61.9	273.8 ± 105.3	232.8 ± 88.8	125.4 ± 51.4	<0.001
Subretinal fluid,	13 (31.7%)	22 (73.3%)	79 (86.8%)	115 (93.5%)	23 (42.6%)	<0.001
Fibrovascular RPED	34 (85.0%)	24 (80.0%)	74 (81.3%)	107 (87.0%)	43 (79.6%)	0.682
Serous RPED	3 (7.3%)	5 (16.7%)	17 (18.7%)	38 (30.9%)	17 (31.5%)	0.009
Subretinal fibrosis	38 (92.7%)	9 (30.0%)	25 (27.5%)	38 (30.9%)	22 (40.7%)	<0.001
Atrophy of ellipsoid zone	12 (29.3%)	6 (20.0%)	24 (26.4%)	28 (22.8%)	5 (9.3%)	0.112

CNV, choroidal neovascularization; MCV, microaneurysmal choroidal vasculopathy; PCV, polypoidal choroidal vasculopathy; RAP, retinal angiomatous proliferation; RPED, retinal pigment epithelial detachment.

^†^Subretinal fibrosis was defined as a fibrous plaque/disciform scarring by means of OCT.

Continuous variables are reported as mean ± standard deviation values. All other data are n (%).

### Treatment outcomes

**Figure 1:**
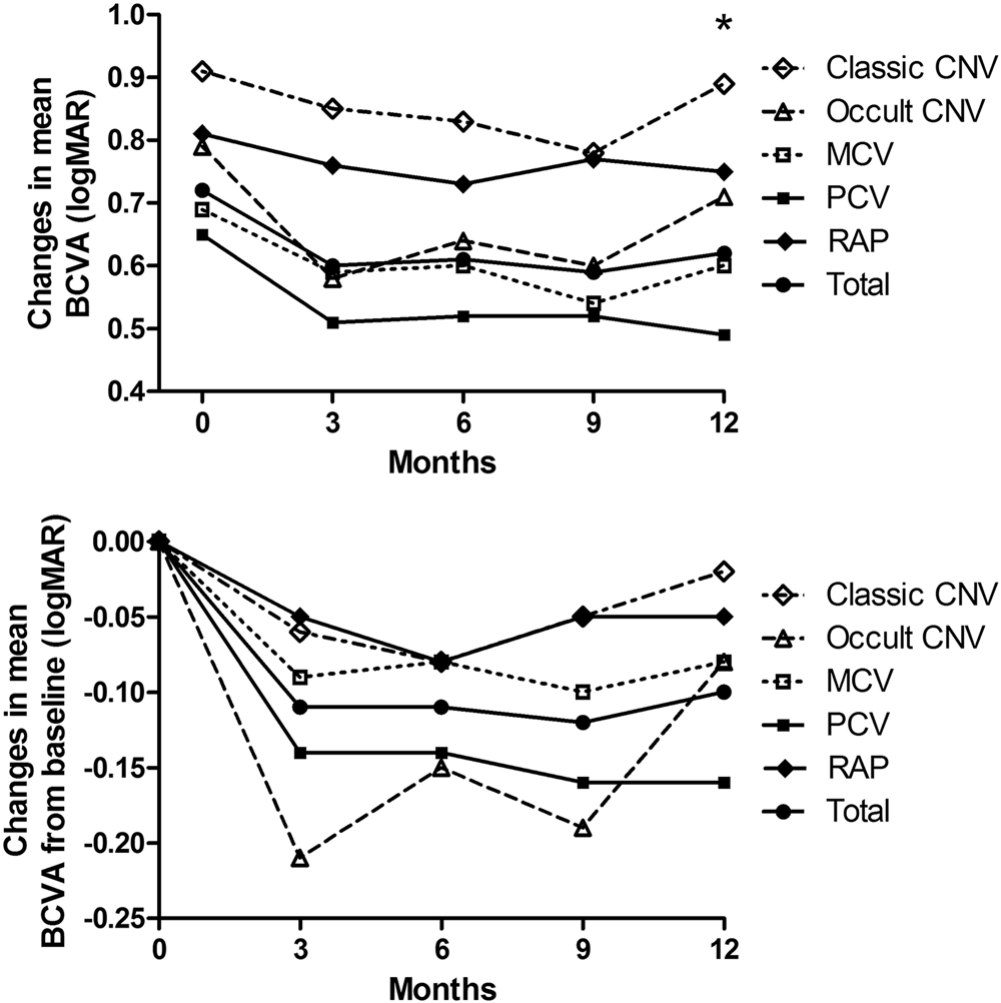
Changes in the mean best-corrected visual acuity (BCVA) of eyes with neovascular age-related macular degeneration subtypes according to the fluorescein angiography and indocyanine green angiography at 3, 6, 9, and 12-month examinations. Results were analyzed only from the patients treated for at least 12 months. There were no significant differences at baseline between the 5 groups, although there was a significant difference at the final time point (*asterisk*). (CNV = choroidal neovascularization; MCV = microaneurysmal choroidal vasculopathy; PCV = polypoidal choroidal vasculopathy; RAP = retinal angiomatous proliferation; *P < 0.05).

**Figure 2:**
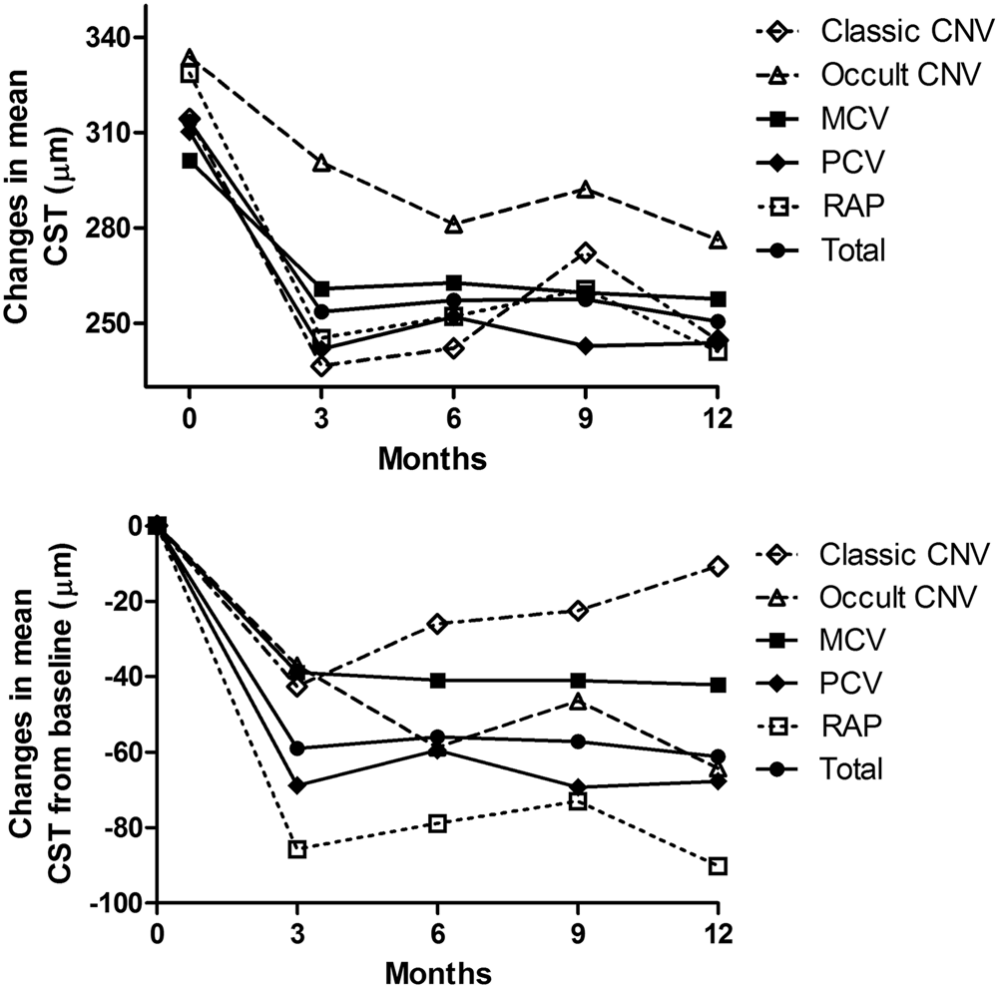
Changes in the mean central macular subfield thickness (CST) of eyes with neovascular age-related macular degeneration subtypes according to the fluorescein angiography and indocyanine green angiography at 3, 6, 9, and 12-month examinations. Results were analyzed only from the patients treated for at least 12 months. (CNV = choroidal neovascularization; MCV = microaneurysmal choroidal vasculopathy; PCV = polypoidal choroidal vasculopathy; RAP = retinal angiomatous proliferation).

Among the study participants, 205 eyes from 191 patients regularly visited the clinic and were treated for at least 12 months. There was no difference in treatment modality including anti-VEGF treatments and PDT between the 5 groups, however, there was a significant difference in final BCVA at 12 months among classic CNV, occult CNV, MCV, PCV, and RAP (20/153, 20/105, 20/80, 20/62, 20/111, respectively, P = 0.046) (Table 3, Fig. 1, Supplemental table 1). The CST at baseline and final visit was not significant among the subtypes (Fig. 2).

**Table 3:** Treatments and 12-month outcomes of neovascular age-related macular degeneration by angiographic subtypes.

Variables	Classic CNV n = 14	Occult CNV n = 20	MCV n = 60	PCV n = 76	RAP n = 35	*P*-value
Mean numbers of PDT	0	0.20 ± 0.41	0.22 ± 0.52	0.20 ± 0.52	0.09 ± 0.28	0.395
Mean numbers of anti-VEGF treatment	4.29 ± 3.12	5.40 ± 2.09	4.50 ± 2.53	4.46 ± 2.31	4.37 ± 2.47	0.577
BCVA, baseline, Snellen (logMAR)	20/166 (0.91)	20/125 (0.79)	20/100 (0.69)	20/90 (0.65)	20/133 (0.81)	0.385
BCVA, 12 months, Snellen (logMAR)	20/153 (0.89)	20/105 (0.71)	20/80 (0.60)	20/62 (0.49)	20/111 (0.75)	0.046
CST, baseline, μm	314.4 ± 133.3	333.8 ± 109.8	301.2 ± 90.1	310.3 ± 94.1	328.7 ± 101.3	0.637
CST, 12 months, μm	244.6 ± 73.7	276.2 ± 117.9	257.6 ± 67.9	243.8 ± 66.9	241.1 ± 56.3	0.391

CNV, choroidal neovascularization; MCV, microaneurysmal choroidal vasculopathy; PCV, polypoidal choroidal vasculopathy; RAP, retinal angiomatous proliferation; SD, standard deviation; VEGF, vascular endothelial growth factor; BCVA, best-corrected visual acuity; CST, central subfield thickness.

Continuous variables are reported as mean ± standard deviation values. All other data are n (%).

## Discussion

The use of FA and ICGA in neovascular AMD has been a clinically relevant issue under investigation since its introduction^14^. Using ICGA has allowed providers to define subtypes of neovascular AMD according to the angiographic characteristics of CNV. However, we still see patients with unclear diagnoses regardless of advanced imaging technology. In this study, neovascular AMD was classified into five subtypes, including the novel MCV group, which was not present in the previous classification system.

This study provides several important issues in investigating the neovascular AMD. We can estimate the proportions of each subtype of neovascular AMD among the Asian population. When classified using FA only, the proportions of classic CNV and occult CNV were 31.0% and 69.0%, respectively. This incidence is similar to previous study, in which classic CNV was found in 23% of eyes and occult CNV in 71%^15^. In contrast, when using ICGA, the most common types of neovascular AMD were PCV (36.3%) and MCV (26.8%). Classic CNV was identified in 12.1% of eyes, occult CNV in 8.8%, and RAP in 15.9%. The sum of eyes with MCV and PCV exceeded 60% of all eyes with neovascular AMD. However, the proportion of RAP was similar to that of previous studies^16,17^. According to the criteria of the EVEREST study^18^, MCV should belong to the category of PCV. However, it could have been classified as a part of occult CNV or chronic CSC in the preceding studies^12,19^, as there is no definite ‘visible’ polyp observed on ICGA. This finding may explain the discrepancy in the proportion of PCV across studies, even those focusing on the same races^20^. In this regard, this novel classification system may contribute to improved categorization of neovascular AMD patients. This method is strengthened by its comparatively high ratio of inter-observer correlation.

Another advantage of this study is that we can compare the proportion of subtypes classified using FA and ICGA, respectively. Of the classic CNV patients diagnosed using FA, only 39.0% were proper classic CNV, compared to just 12.8% in the occult CNV. While FA was useful to assess leakage from CNV lesions, it was insufficient for demarcating occult or poorly defined CNV, which is necessary for identifying PCV and MCV. Yannuzzi *et al*. reported that the prevalence of RAP is between 10% and 15%, with a female and Caucasian predominance^16^. The frequency of RAP in this study (15.9%) is consistent with findings reported in similar studies on Caucasians, but is higher than that reported in Asian populations (4.5%)^21,22^. Lee *et al*.^23^ also reported that the rate of RAP was high in Koreans (25%). Interracial differences in the proportion of neovascular AMD can be expected, because the Y402H variant plays a major role in the etiology of AMD and is obviously different between Caucasians and Asians^24^. Most Korean patients do not have the Y402H variant. Therefore, there are likely other, yet unidentified, genetic variants that also promote disease progression in RAP.

**Figure 3:**
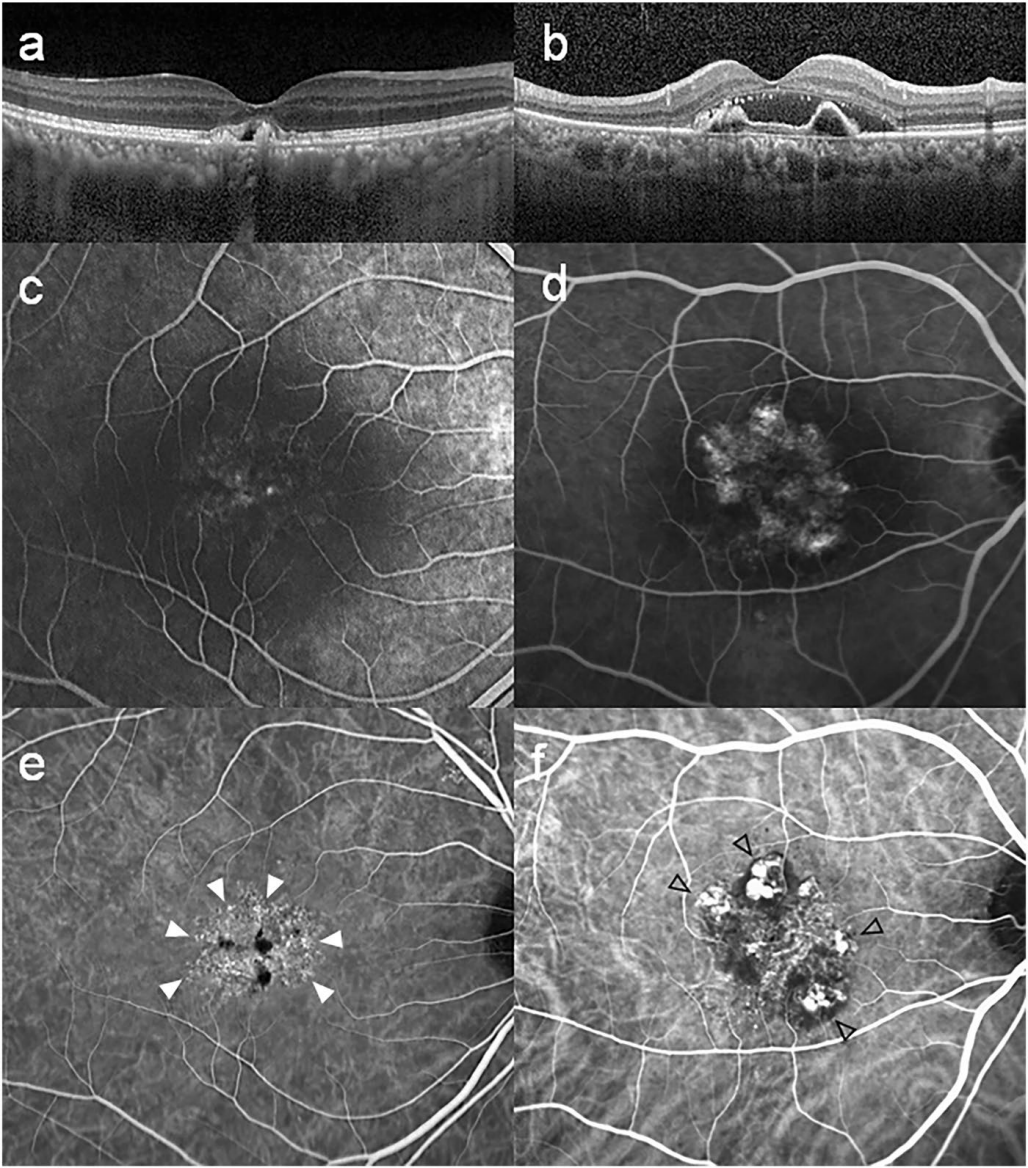
Multimodal imaging of a 53-year-old male with microaneurysmal choroidal vasculopathy (MCV, left column) and 66-year-old male with polypoidal choroidal vasculopathy (PCV, right column). (**a**) Spectral domain optical coherence tomography (OCT) showing pigment epithelial elevation overlying a markedly thickened choroid and dilated choroidal vessels with minimal subretinal fluid. (**b**) Moderately thickened choroid is shown on OCT, and there is a discrete polypoidal structure between retinal pigment epithelium and Bruch’s membrane. (**c**,**d**) Early fluorescein angiography showing leakage from the choroidal neovascularization corresponding to the hyperfluorescent spots in indocyanine green angiography (ICGA). (**e**) ICGA in the early phase showing branching vascular network. Note that there is no definite polyp, other than small aneurysmal dilations (white arrow heads). (**f**) ICGA demonstrates multiple hyperfluorescent spots around the fovea corresponding to the polyps (opened black arrow heads) of PCV.

Sometimes there are ambiguous features on angiography, and retinal manifestations of PCV resemble those of chronic CSC or occult CNV^12,19^. Moreover, Uzawa *et al*.^25^ and Tan *et al*.^26^ advocated that the PCV is not a homogenous disease entity, but a spectrum of disease with discriminative characteristics. Previous study also revealed a lower anti-VEGF treatment response associated with thicker choroids in PCV patients^27^. These results are likely to be caused by the coexistence of different subtypes within the category we classify as PCV. In this regard, the refined classification system including MCV may reduce such debate and clarify the subtype of neovascular AMD. MCV is distinct from PCV by the absence of definite polyps. It is distinct from occult CNV by the presence of microaneurysms, which are connected with a lacy choroidal vasculature on ICGA (Fig. 3). The choroid of the MCV group patients was most thickened, and the baseline BCVA was the best among the subtypes. However, the MCV group only had one line of visual gain after 12 months of anti-VEGF treatment, which was inferior to that of the PCV group. These results suggest that the MCV group has its own characteristics that distinguish it from other groups.

The characteristics of the patients in MCV group, which includes male predominance, thickened choroid, alterations in RPE layer, and relatively good visual acuity at baseline, is quite interesting because it is comparable to CSC. Freund and associates have proposed the existence of a pachychoroid-driven spectrum of disease comprising “CSC, pachychoroid pigment epitheliopathy, and pachychoroid neovasculopathy,” which may support the hypothesis that CSC develops Type 1 neovascularization^28^. They demonstrated that it is a thickened choroid that drives the RPE changes seen in these eyes. In this regard, the features of MCV may correspond to the pachychoroid spectrum disease and its relevance seems even stronger than PCV. Given that choroid was most thickened in MCV group patients, it is possible that multiple microaneurysms along with the BVN is dominated by changes in the RPE, which, in tum, may be secondarily related to the chronicity of choroidal vascular hyperpermeability. However, since this study is a cross-sectional study, it is difficult to identify the causal relationship between MCV and pachychoroid spectrum diseases. Further longitudinal research is needed in this regard.

There were several limitations in the present study. First, because of its retrospective nature, using data from a single tertiary hospital, our results may not be generalizable to the overall Korean population and microaneurysms of MCV subtype could not be identified histologically. A second limitation is that the classification was applied at the time of the review process; therefore, it did not influence the treatment regimens. This may be the reason why there was no difference in treatment modality. Another limitation of this study is its relatively short follow-up period of 12 months. Our results, therefore, may not reflect the final outcomes of neovascular AMD. Despite these limitations, our data provide evidence that are compared to results from other American, Asian, and even previous Korean studies. We observed five subtypes of neovascular AMD in this study, including classic CNV, occult CNV, MCV, PCV, and RAP. The demographic, angiographic and OCT findings varied depending based on these subtypes. The MCV group has distinctive features from other groups. Further investigation is needed to clarify the pathogenesis and prognosis of this subtype of neovascular AMD.

## Methods

### Design and setting

We conducted a review of patients at a single center according to the tenets of the Declaration of Helsinki. The study protocol was approved by the institutional review board and ethics committees at Kyung Hee University Hospital. All patients provided written informed consent before the treatment.

The study population consisted of consecutive treatment naïve patients aged 50 years or older who were diagnosed with neovascular AMD between January 2011 and June 2017. The exclusion criteria were as follows: eyes with myopic CNV, central serous chorioretinopathy (CSC), diabetic retinopathy, retinal vasculopathy, inflammatory diseases, intraocular tumors, history of intraocular surgery (other than cataract surgery), and other ocular diseases that could influence visual function.

Our baseline investigations were as follows: an examination for the Snellen best-corrected visual acuity (BCVA) and anterior segment by slit lamp; dilated fundus examination; and confocal scanning laser ophthalmoscopy (SLO) FA and ICGA using eye-tracked Spectralis Heidelberg Retina Angiograph (HRA) + OCT (Heidelberg Engineering, Heidelberg, Germany). Angiograms were obtained using a standardized imaging protocol. The early phases (up to 60 s) were imaged using dynamic ICGA and FA. Consecutive photographs were then taken for up to 1 min, and again at approximately 2.5, 5, 10, 15 and 20 minutes. These images were reviewed using Heidelberg Eye Explorer software (V.1.7.0.0). We also obtained simultaneous SD-OCT cross-sectional images, which correspond to either the site of the hot spot, plaque, or the network vessels on SLO. In addition, horizontal and vertical cross-sectional enhanced depth imaging (EDI)-OCT was performed at the fovea.

### ICGA and FA-based classification

**Figure 4:**
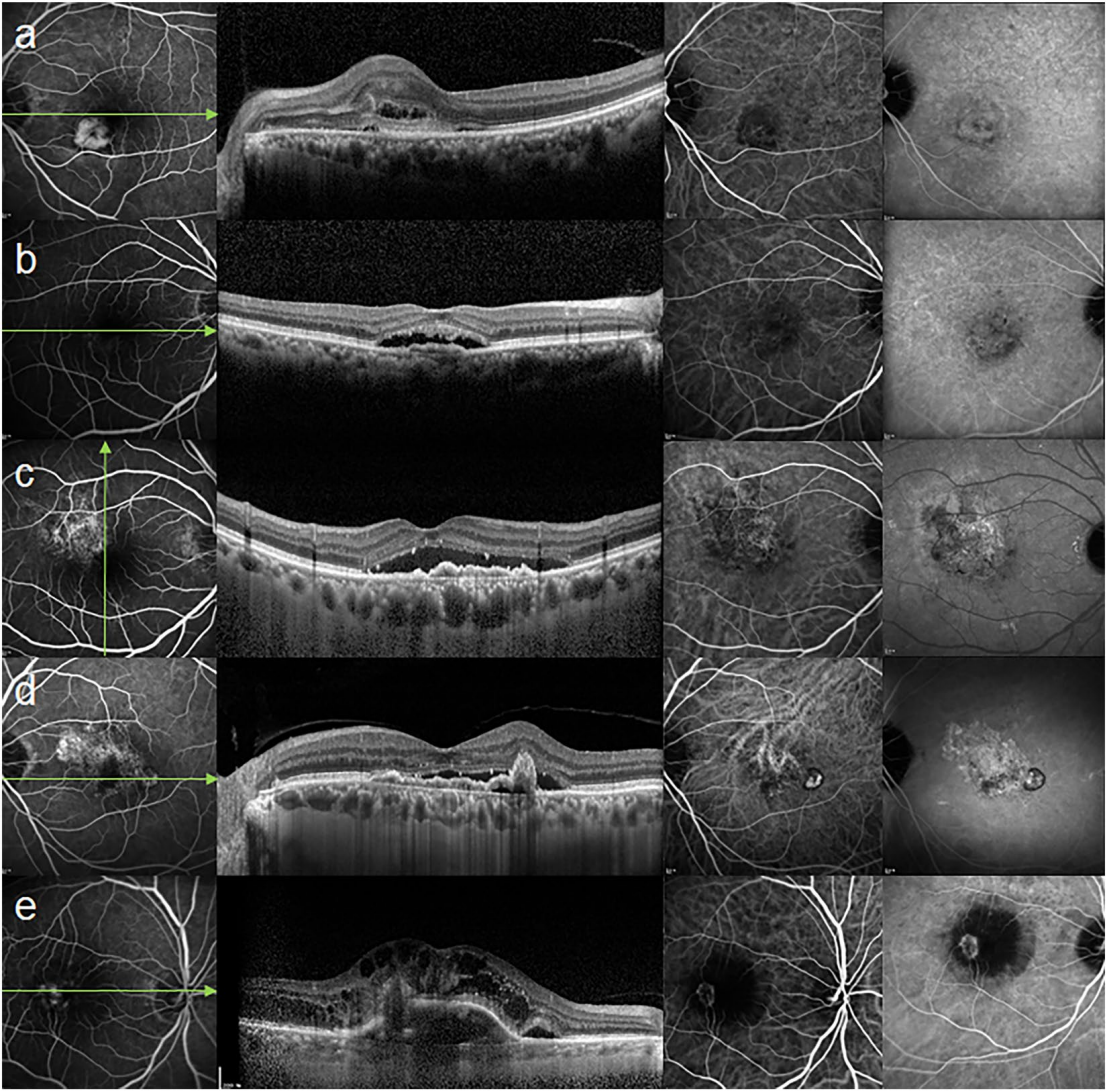
Representative images of neovascular age-related macular degeneration subtypes, illustrating features on fluorescein angiography (FA), optical coherence tomography (OCT), and indocyanine green angiography (ICGA). (**a**) Classic choroidal neovascularization (CNV): early FA showing leakage from CNV. (**b**) Occult CNV: there is indistinct leakage in the early phase FA, which was not accompanied by abnormal vasculature other than plaques in the late phase of ICGA. (**c**) Microaneurysmal choroidal vasculopathy: early FA showing leakage from the branching vascular network (BVN). There is no definite polyp, other than small aneurysmal dilations, on ICGA. Choroidal thickening accompanied by irregular flat retinal pigment epithelium detachment is shown on OCT. (**d**) Polypoidal choroidal vasculopathy: ICGA demonstrates polyps at the periphery of a large BVN. (**e**) Retinal angiomatous proliferation: FA and ICGA showing retina-to-choroidal anastomosis compatible with the area of CNV on OCT.

We devised a classification system of neovascular AMD based on prior observation of patients from our clinic. Two independent retinal specialists (K.B. & E.S.K.) who were blinded to the treatments classified the angiographic findings into one of five types (Fig. 4). In cases in which there was disagreement between the two observers, a third independent blinded observer (S.Y.Y.) analyzed the images. When it was difficult to specify one of these categories, it was classified as miscellaneous. Angiographic classifications were defined as below:

- Classic CNV was defined by discrete, well-demarcated focal areas of hyperfluorescence that can be discerned in the early phases of the FA. Hyperflourescence increases in intensity and extends beyond the boundaries of the hyperfluorescent area, which is identified in earlier phases of the angiogram of mid- and late-phase frames.
- Occult CNV was defined by late choroidal-based leakage in the early or mid-phase FA to account for an area of leakage in the late phase. There was no clearly identifiable classic CNV in FA or the presence of a polypoidal lesion, vascular network, or anastomosis in ICGA.
- Microaneurysmal choroidal vasculopathy (MCV) was defined by the presence of early multiple focal hyperfluorescent areas (appearing within the first 6 minutes after injection of indocyanine green) in ICGA. These areas consist of microaneurysms, which are smaller than the secondary branch of major retinal vein width, and a branching vascular network (BVN) that fills simultaneously during the choroidal filling phase.
- Polypoidal choroidal vasculopathy (PCV) was defined by the presence of early subretinal focal ICGA hyperfluorescence with a definite polyp. In addition, there was at least one of the following angiographic or clinical criteria: (i) association with a BVN; (ii) nodular appearance when viewed stereoscopically; (iii) presence of a hypofluorescent halo (in first 6 minutes); (iv) orange subretinal nodules on a stereoscopic color fundus photograph (polyp corresponding to ICGA lesions); or (v) association with massive submacular hemorrhage (defined by a hemorrhage of at least four disk areas).
- Retinal angiomatous proliferation (RAP) was defined by the subretinal, intraretinal, or preretinal hemorrhages or by retinal edema, dilated retinal vessels, retinal–retinal anastomosis, sudden termination of the retinal vessels, a ring of lipid exudates and PED, and retinal–choroidal anastomosis in dynamic FA and ICGA.

### Treatment protocols

Patients were treated using anti-VEGF agents (Bevacizumab, Ranibizumab, and Aflibercept) by two retinal specialists (E.S.K. and S.Y.Y.) using a standardized treat-and-extent protocol^29^. We defined patients in whom subretinal or intraretinal fluid was present or increased, even in the presence of monthly anti-VEGF injections, as non-response. Active polyps, as well as lesions that were non-responsive to 3 consecutive anti-VEGF injections, were treated using photodynamic therapy (PDT) with verteporfin (Visudyne, Novartis International AG, Basel, Switzerland) guided by ICGA. Full-dose, full-duration PDT was performed. The treatment zone only included the visible polyps in PCV and active CNV lesions in other categories. The treatments were initiated or repeated when there were clinical signs of disease activity, such as a drop in BCVA of ≥1 line, subretinal fluid or macular edema, or evidence of active CNV on angiography.

### Study measurements

We recorded the patient age, sex, BCVA, and the lesion type at the time of the first injection by ICGA. During the follow-up visits at 3, 6, 9, and 12 months after the initial treatment, all patients underwent BCVA measurement, dilated fundus examination, and SD-OCT. At each visit, all of the treatments were recorded, along with the BCVA and central subfield thickness of the macula.

The following parameters were used to evaluate the SD-OCT images: the presence and type of RPE detachment, subretinal fibrosis, atrophy of the ellipsoid zone, and subfoveal choroidal thickness.

### Study outcomes

The primary outcome was the difference in the baseline characteristics of the subtypes of neovascular AMD based on novel classification. The secondary outcomes included the mean BCVA and central subfield thickness 12 months after initiating treatment, and the number of intravitreal injections.

### Statistical analysis

Statistical analyses were performed using SPSS version 24 (SPSS, Chicago, Illinois, USA). Interobserver agreements were evaluated using the intraclass correlation coefficient (ICC) value. The Snellen BCVA was converted into logarithm of the minimal angle of resolution (LogMAR) units prior to the analysis. The chi-square test was used to compare categorical variables, while the one-way analysis of variance (ANOVA) was used for continuous variables. Appropriate parametric analyses were performed when the data had a normal distribution. P-values less than 0.05 were considered statistically significant.

## Supplementary information

Supplementary table 1
