# Supplementary material for: Angiographic Subtypes of Neovascular Age-related Macular Degeneration in Korean: A New Diagnostic Challenge
Source: Sci Rep. 2019 Jul 4;9:9701. doi: 10.1038/s41598-019-46235-3 (PMC6609644; doi:10.1038/s41598-019-46235-3)
Supplement: Supplementary file 1 — Supplementary table 1 [file 41598_2019_46235_MOESM1_ESM.docx]

**Angiographic Subtypes of Neovascular Age-related Macular Degeneration in Korean: A New Diagnostic Challenge**

Kunho Bae,^1^ Sung Rae Noh,^2^ Se Woong Kang, MD,^3^ Eung Suk Kim,^2^ Seung-Young Yu^2^

^1^Department of Ophthalmology, Dongguk University, Ilsan Hospital, Goyang, South Korea.

^2^Department of Ophthalmology, Kyung Hee University Hospital, Kyung Hee University, Seoul, South Korea.

^3^Department of Ophthalmology, Samsung Medical Center, Sungkyunkwan University School of Medicine, Seoul, South Korea.

**Supplemental Table 1.** Visual and functional outcomes of microaneurysmal choroidal vasculopathy (MCV) and polypoidal choroidal vasculopathy (PCV) patients according to the combined photodynamic therapy (PDT).

| **Variables** | **MCV** | | ***P*-value** | **PCV** | | ***P*-value** |
| --- | --- | --- | --- | --- | --- | --- |
|  | **No PDT** | **With PDT** |  | **No PDT** | **With PDT** |  |
| BCVA, baseline, mean, Snellen | 0.72 | 0.56 | 0.390 | 0.62 | 0.81 | 0.372 |
| BCVA, 12 months, mean, Snellen | 0.60 | 0.59 | 0.962 | 0.50 | 0.47 | 0.873 |
| CST, baseline, mean, μm | 296.8 | 321.1 | 0.539 | 309.7 | 313.3 | 0.900 |
| CST, 12 months, mean, μm | 257.8 | 256.4 | 0.946 | 245.2 | 236.6 | 0.656 |
| △BCVA, mean, Snellen | 0.11 | -0.03 | 0.159 | 0.12 | 0.34 | 0.246 |
| △CST, mean, μm | 41.4 | 45.7 | 0.918 | 66.1 | 76.7 | 0.725 |
| Mean numbers of anti-VEGF | 4.43 | 4.82 | 0.602 | 4.38 | 4.91 | 0.516 |

BCVA, best-corrected visual acuity; CST, central subfield thickness; VEGF, vascular endothelial growth factor

Independent t-test was used for all variables.
